# Rapidly Solidified Aluminium Alloy Composite with Nickel Prepared by Powder Metallurgy: Microstructure and Self-Healing Behaviour

**DOI:** 10.3390/ma12244193

**Published:** 2019-12-13

**Authors:** Alena Michalcová, Anna Knaislová, Jiří Kubásek, Zdeněk Kačenka, Pavel Novák

**Affiliations:** Department of Metals an Corrosion Engineering, Faculty of Chemical Technology, University of Chemistry and Technology, Prague 16628, Czech Republic; knaisloa@vscht.cz (A.K.); kubasekj@vscht.cz (J.K.); kacenkaz@vscht.cz (Z.K.); panovak@vscht.cz (P.N.)

**Keywords:** self-healing, aluminium alloy, microstructure

## Abstract

Composite material prepared by spark plasma sintering (SPS) from a powder mixture of AlCrFeSi rapidly solidified alloy and 5 wt. % of Ni particles was studied in this work. It was proven that during SPS compaction at 500 °C, no intermetallic phases formed on the surface of Ni particles. The material exhibited sufficient mechanical properties obtained by tensile testing (ultimate tensile stress of 203 ± 4 MPa, ductility of 0.8% and 0.2% offset yield strength of 156 ± 2 MPa). Tensile samples were pre-stressed to 180 MPa and annealed at 450 and 550 °C for 1 h. Annealing at 450 °C did not lead to any recovery of the material. Annealing at 550 °C caused the full recovery of 0.2% offset yield strength, while the ductility was decreased. The self-healing behaviour originates from the growth of intermetallic phases between the Ni particle and the Al matrix. The sequence of NiAl, Ni_2_Al_3_ and NiAl_3_ intermetallic phases formation was observed. In particular, the morphology of the NiAl_3_ phase, growing in thin dendrites into the Al matrix, is suitable for the closing of cracks, which pass through the material.

## 1. Introduction

Self-healing materials are designed in order to enrich structural materials by the ability of closing crack that was formed during material utilization. Closing of the crack will lead to the restoration of the mechanical cohesion of the material. This may decrease the impacts of an accident caused by material failure.

Several mechanisms of the self-healing behaviours have been proposed. Precipitation mechanism at low temperature was observed in an Fe-Au alloy [1]. In several other alloys, precipitation self-healing of the crack occurred after an exposure to elevated temperature [2,3,4,5]. Phase transformation may also be a driving force for the self-healing behaviour. Composite materials with the shape memory alloy (SMA) reinforcement and the matrix with off-eutectic composition were described in [6,7]. In this case, the phase transformation of the SMA (smart material, in this case shape memory alloy) reinforcement leads to the crack closing and a partial matrix melting heals the already closed crack. Another type of the self-healing behaviour leading to crack closing was observed in Al-Ag alloy, where the phase transformation was between the Ag_2_Al phase and the fcc-Al [8]. A different strategy for the crack healing is a usage of an encapsulated healing agent. A low melting metal or alloy is filled into a hollow diffusion barrier, typically composed of oxides [5]. The idea is that the spreading crack will break the diffusion barrier and enter the area with the low melting healing agent. After annealing, the low melting healing agent will fill the crack and the hollow diffusion barrier remains as a spherical pore in the microstructure. 

The idea presented in this paper is to modify the encapsulated-healing-agent strategy by adding Ni powder into rapidly solidified Al alloy. The diffusion barrier will be formed by nickel aluminides and may provide the material with a better cohesion, compared to the oxides. During annealing, intermetallic phases will be formed by a reaction of Ni and Al. This process is accompanied by volume changes [9,10], so a decreased level of porosity may be expected compared to the self-healing by the encapsulated low-melting metal/alloy mechanism. By this innovative process, self-healing materials will be prepared by the two-step powder metallurgy processing (1) preparation of rapidly solidified alloy, (2) compaction. No further steps are necessary to supply self-healing agent to the material. Simple powder mixing before compaction is required.

## 2. Materials and Methods 

Powder alloy with intended composition AlCr6Fe2Si1 (given in wt. %) was prepared by a gas atomisation and mixed with 5 wt. % of a gas atomized Ni. In both cases, the finest granulometric fraction of the size less than 63 µm was used. The powder mixture was consolidated using the spark plasma sintering (SPS) device (FCT Systeme HP D 10, Rauenstein, Germany) forming a bulk sample of 40 mm in diameter and approximately 13 mm in height. Chemical composition measured by X-Ray fluorescence spectroscopy (XRF, ARL 9400 XP, Thermo ARL, Switzerland) of the sintered sample denoted as “AlCrFeSi + Ni” is given in Table 1.

Bulk material was observed by scanning electron microscopy (SEM, TESCAN VEGA 3 equipped by energy dispersive spectroscopy (EDS) detector by Oxford Instruments, Abingdon, UK). SEM samples were prepared by mechanical grinding (P320-P4000), by polishing using diamond paste (D3, D0.7) and by etching in 0.5 % HF solution. TEM samples were prepared by ion polishing using a Gatan PIPs device (Gatan, Pleasanton, CA, USA). Observation by the transmission electron microscopy (TEM) was done by Jeol 2200 FS equipped by EDS detector by Oxford Instruments. Tensile testing was performed by the Instron 5882 universal loading machine with the strain rate of 1 mm / min. Dog-bone shaped samples with the length of 16 mm, width of 4 mm, and height of 2 mm with 20 mm radius were used. Phase composition was determined by XRD using diffractometer PANanalytical X´Pert PRO, Co lamp (Almelo, The Netherlands).

## 3. Results and Discussion

### 3.1. Microstructure of As-Sintered AlCrFeSi + Ni Material

**Figure 1:**
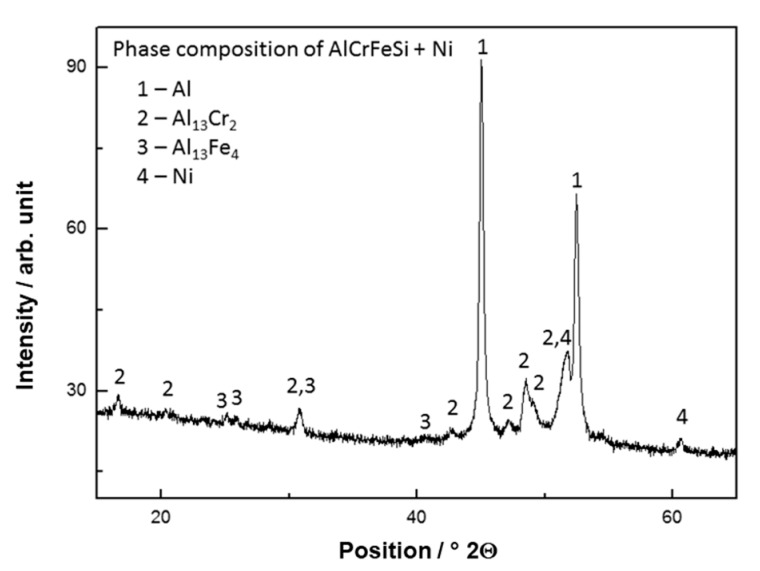
X-Ray diffraction pattern of as-sintered AlCrFeSi + Ni.

Bulk sintered material was composed of Al matrix, Al_13_Cr_2_ and Al_13_Fe_4_ intermetallic phases and Ni as proven by XRD pattern, shown in Figure 1.

**Figure 2:**
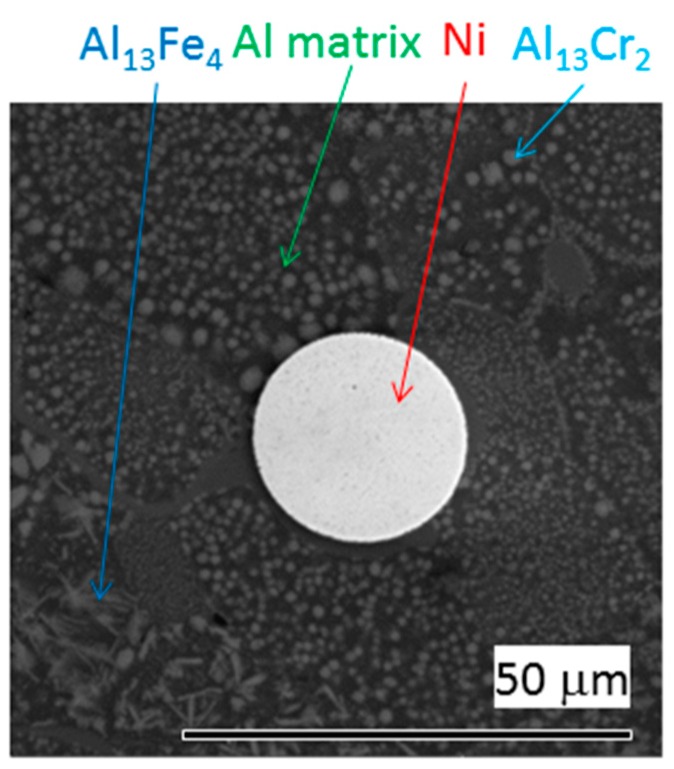
Micrograph of as-sintered AlCrFeSi + Ni (SEM/BSE).

Figure 2 shows the SEM micrograph of the as-sintered AlCrFeSi + Ni composite material. In the Al matrix, fine particles of intermetallic phases Al_13_Cr_2_ (round) and Al_14_Fe_4_ (needles) are visible. The initial particles of Al alloy were deformed during the SPS process, while Ni particles remained spherical.

**Figure 3:**
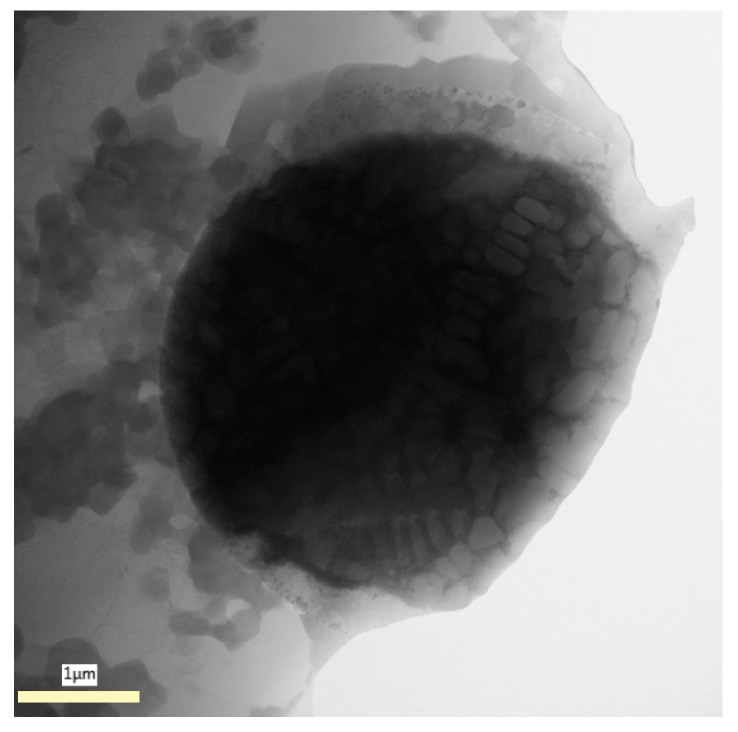
Detailed micrograph of as-sintered AlCrFeSi + Ni (TEM).

To describe the boundary between the Al matrix and the Ni particle, it is necessary to use the TEM. The TEM micrograph is given in Figure 3. The light grey Al matrix contains fine intermetallic particles. The internal structure of the Ni particle is dendritic.

**Figure 4:**
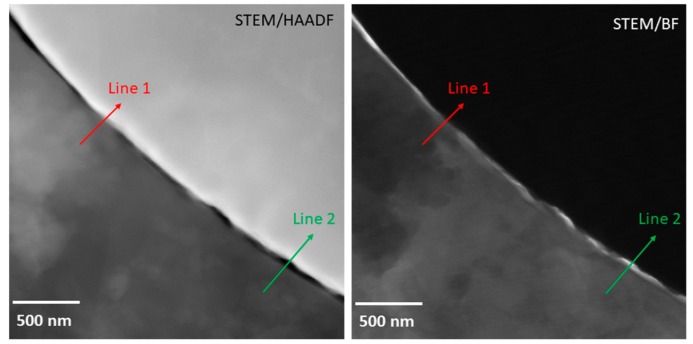
Detailed micrograph of boundary between Al matrix and Ni particle in the as-sintered AlCrFeSi + Ni (STEM) with indication of line profiles.

More detailed view was obtained in STEM mode (Figure 4). Sufficient contact of the Al matrix with the Ni particle was observed, but the connection was not uniform. EDS line profiles were measured in a location with the good connection (Line 1) and in a location where the gap is evident (Line 2).

**Figure 5:**
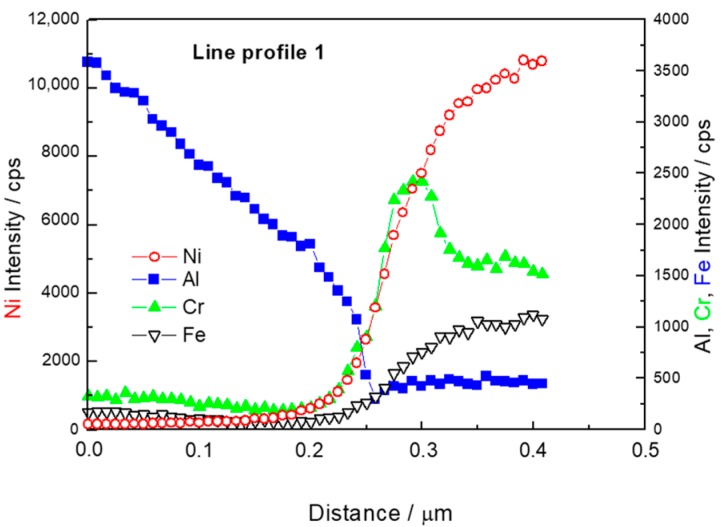
Line profile 1, location shown in Figure 4.

The EDS profile of the Line 1 is plotted in Figure 5. Although it is the location with a good connection, no plateau in any profile was observed. This means that no intermetallic phase with a stoichiometric composition was formed. Enrichment of the Ni particle surface by Cr was observed. This impurity might originate from the initial Ni powder.

**Figure 6:**
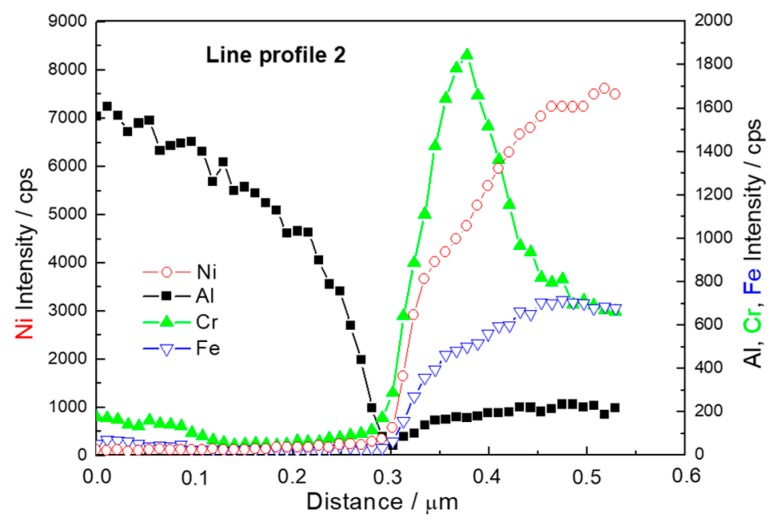
Line profile 2, location shown in Figure 3.

The EDS profile of the Line 2 is plotted in Figure 6. At the position of 0.3 µm from the beginning of the line, the minimum of all the elements’ profile lines can be seen. This is the position of the gap. No plateau was observed again in the vicinity of the gap. This proves that the sintering process caused only partial connection of Al matrix and Ni particles. The other possible explanation of the presence of a gap would be a formation of the gap caused by different thermal expansion of intermetallic interlayer between Al matrix and Ni particle during cooling of sintered samples. As the composition changes gradually (Figure 5), there is no evidence of phase transformation during the SPS process. 

### 3.2. Microstructure of AlCrFeSi + Ni Material Annealed at 550 °C for 1 h

**Figure 7:**
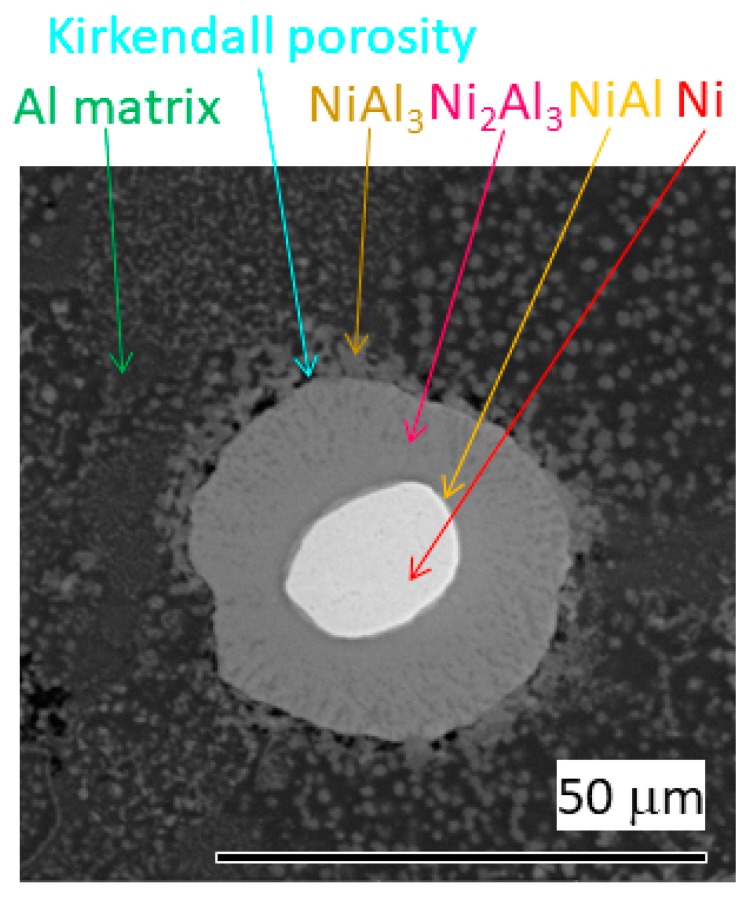
Micrograph of AlCrFeSi + Ni annealed at 550 °C for 1 h (SEM/BSE).

Figure 7 illustrates microstructure of AlCrFeSi + Ni composite after annealing at 550 °C for 1 h. The composite behaved like diffusion couple that was already well described in several works [10,11,12,13,14,15,16]. The Ni residual core is covered by a thin layer of NiAl phase. A large layer of Ni_2_Al_3_ is on the top of the NiAl layer. Dendrites of NiAl_3_ grow into Al matrix. Porosity caused by diffusion (Kirkendall porosity) can be observed between NiAl_3_ dendrites. The intermetallic particle has an irregular shape compared to the initial Ni sphere. This observation verifies the volume change during formation of intermetallic phases, which is a sign of the self-healing behaviour potential of the AlCrFeSi + Ni material.

**Figure 8:**
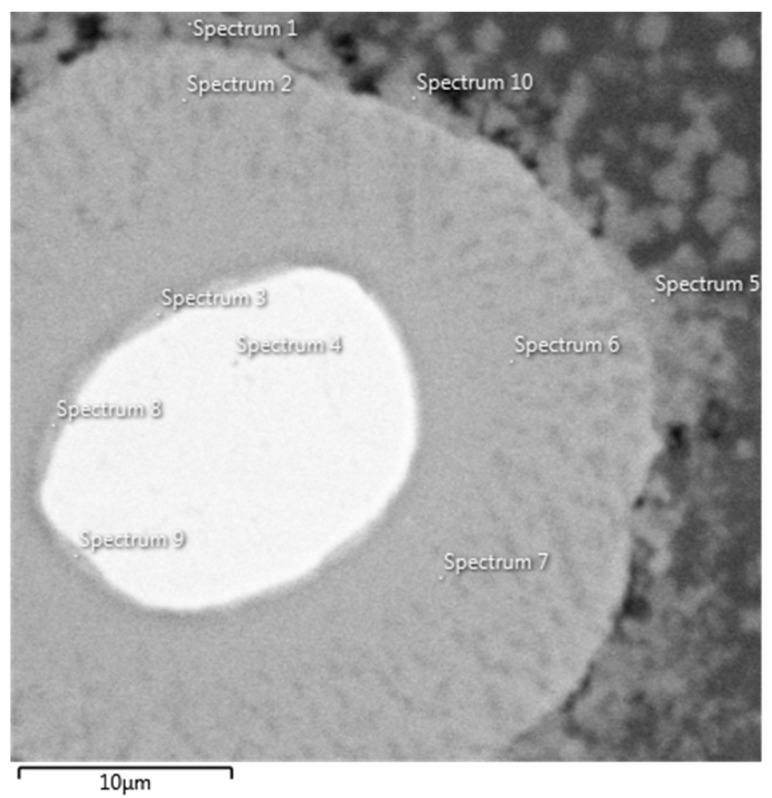
Micrograph of AlCrFeSi + Ni annealed at 550 °C for 1 h with labelled EDS point spectra.

The phase composition was determined by point EDS analysis. Actual positions of each measured spectrum are shown in Figure 8. Theoretical content of Al in NiAl is 50 at. %, in Ni_2_Al_3_ 60 at. % and in NiAl_3_ 75 at. %. The values of Al content presented in Table 2 are slightly higher that the teoretical ones. It is due to interaction volume of the EDS point spectra measurement (several cubic micrometers). It is highly probable that the EDS spectra contain a partial signal from the Al matrix, which increases the measured values.

### 3.3. Tensile Testing

**Figure 9:**
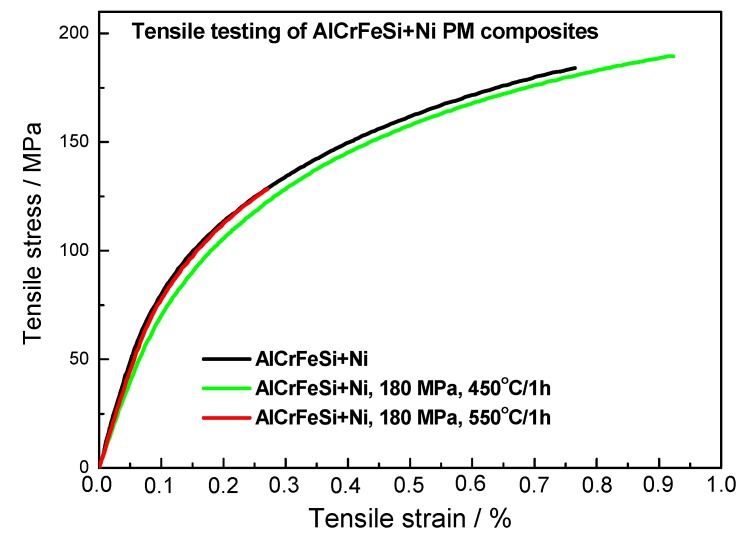
Tensile testing of AlCrFeSi + Ni, black—tensile tested in as-sintered state, green—prestressed up to 180 MPa, annealed at 450 °C / 1 h and tensile tested, red—prestressed up to 180 MPa, annealed at 550 °C/1 h and tensile tested.

Figure 9 plots the results of tensile testing of AlCrFeSi + Ni composite material. The black curve corresponds to a testing of the material in the as-sintered state. The results give the essential description of behaviour of the material. The ultimate tensile stress was 203 ± 4 MPa, the ductility was 0.8 %. The yield stress cannot be estimated clearly, so the 0.2% offset yield strength was measured and it reaches the value of 156 ± 2 MPa. Subsequently, the samples were pre-stressed to 180 MPa, where the tensile test was stopped. The pre-stressed samples were annealed for 1 h at temperatures of 450 °C and 550 °C. The pre-stressed and annealed samples underwent the tensile testing under the same conditions as the as-sintered AlCrFeSi + Ni material. Annealing at 450 °C caused an increase in ductility, but the 0.2% offset yield strength was lower. This means that the most important property form the structural point of view was not preserved. The material annealed at 550 °C had a lower ductility, but the 0.2% offset yield strength was exactly the same as the one of the as-sintered material.

For the self-healing materials with SMA reinforcement, is was described that the material after healing reached 95% of UTS value and the ductility decrease from 6.4% to 2.2% (corresponding to 34% of the original ductility value) [6]. In our case, 63% of UTS value was achieved but what is more important by preserving the value of 0.2% offset yield strength. The value of ductility decreased in our case from 0.8% to 0.27%. This means that the self-healed material had ductility reaching 33% of the value of sintered material, which is in good agreement with other materials with self-healing properties [6].

**Figure 10:**
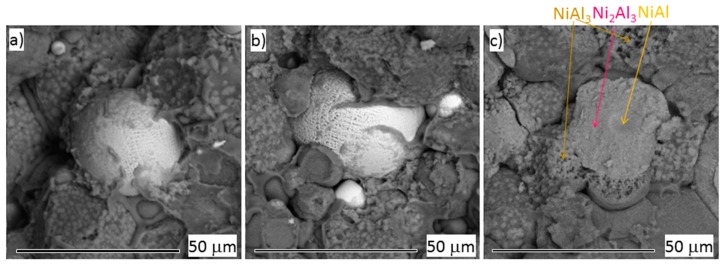
Fractography of AlCrFeSi + Ni, (**a**) tensile tested in as-sintered state, (**b**) prestressed up to 180 MPa, annealed at 450 °C / 1 h and tensile tested, (**c**) prestressed up to 180 MPa, annealed at 550 °C / 1 h and tensile tested (SEM).

Figure 10 shows the SEM micrographs of fracture surfaces of the tensile tested materials. The Ni particle visible in the as-sintered material (a) has a dendritic surface morphology. The SPS process was successful, as the Ni particle is an integral part of the material and does not serve as stress concentrator (the Al matrix is still surrounding the Ni particle). The morphology of the Ni particle after annealing at 450 °C was not changed (b) and the whole fracture surface looks similar to the as-sintered material. The fracture surface of the material annealed at 550 °C is significantly different. The fracture surface of Ni_2_Al_3_ particle is shown in the middle (c) with small round island of NiAl. Dendrites of NiAl_3_ are interconnecting Ni_2_Al_3_ particle with Al matrix in the vicinity. In the areas where initial Ni particles were close (top left corner), the NiAl_3_ are not observed. In these places, polyhedrons of Ni_2_Al_3_ are formed. This may be the reason for the decrease of the ductility of the material annealed at 550 °C. 

## 4. Conclusions

AlCrFeSi + Ni composite material was successfully prepared by the SPS process. During sintering, no intermetallic phase was formed on the surface of Ni particles, as proved by TEM observation. The results of tensile testing showed sufficient mechanical properties of the AlCrFeSi + Ni material (ultimate tensile stress: 203 ± 4 MPa, ductility: 0.8%, 0.2% offset yield strength: 156 ± 2 MPa).

The tensile testing samples were pre-stressed to 180 MPa (the value above 0.2% offset yield strength) and annealed. On the annealed samples, the tensile testing was performed again. The sample annealed at 450 °C, which is under the temperature of intermetallic formation, exhibits a slightly higher ductility, but the 0.2% offset yield strength did not reach the value of the as-sintered material. The value of 0.2% offset yield strength for the sample annealed at 550 °C was similar to the value of the as-sintered material. The self-healing behaviour was enabled by the formation of a sequence of nickel aluminides from initial Ni particle into Al matrix. The formation of Ni_2_Al_3_ polyhedrons caused the decrease of materials ductility. This problem might be solved by an optimization of the Ni content in the material.

## Figures and Tables

**Table 1:** Chemical composition of AlCrFeSi + Ni.

Element (wt. %)	Al	Si	Cr	Fe	Ni
AlCrFeSi + Ni	86.42	1.53	5.47	1.86	4.68
error	0.17	0.06	0.11	0.07	0.11

**Table 2:** Chemical composition of points labelled in Figure 8 given in at. % (EDS).

Element (at. %)	Al	Cr	Fe	Ni	Phase
Spectrum 1	84.15	7.85	2.44	5.55	NiAl_3_
Spectrum 2	75.94	2.66	1.74	19.65	Ni_2_Al_3_
Spectrum 3	51.6	5.75	3.85	38.8	NiAl_3_
Spectrum 4	2.34	10.65	7.57	79.43	Ni
Spectrum 5	83.16	7.27	2.44	7.13	NiAl_3_
Spectrum 6	74.99	2.68	1.77	20.56	Ni_2_Al_3_
Spectrum 7	74.54	2.78	1.54	21.14	Ni_2_Al_3_
Spectrum 8	64.76	4.53	3.00	27.71	NiAl
Spectrum 9	58.93	5.14	3.52	32.41	NiAl
Spectrum 10	82.36	5.61	2.94	9.09	NiAl_3_
